# Navigating ultrasound-guided regional anesthesia: narrative review on techniques, evidence, and evolving clinical practice

**DOI:** 10.1097/MS9.0000000000005041

**Published:** 2026-09-30

**Authors:** Muddassir Khalid, Wadiha Shah, Umair Ali, Asra Amjad, Tooba Khurshid, Sopoma Gosh Ria, Olivia Benny

**Affiliations:** a Department of Medicine, Nishtar Medical University, Multan, Pakistan; b Department of Medicine, Gajju Khan Medical College, Swabi, Pakistan; c Department of Pharmacy, University of Swabi, Swabi, Pakistan; d Department of Medicine, Islamic International Medical College (Riphah International University, Rawalpindi, Pakistan; e Department of Medicine, Karachi Medical and Dental College, Karachi, Pakistan; f Department of Medicine, Mugda Medical College (University of Dhaka), Dhaka, Bangladesh; g Department of Medicine, School of Medicine, Keele University, Keele, UK

**Keywords:** anesthesia, fascial block, neuraxial block, post-operative analgesia, UGRA, ultrasound-guided regional anesthesia

## Abstract

Ultrasound-guided regional anesthesia (UGRA) has emerged as a new technical approach to provide real-time visualization of the needle pathway to minimize the risk of trauma to surrounding structures, notably vessels and nerves. This review will examine the basic concepts of UGRA and describe how regional anesthetic methods are categorized by region. Additionally, it explores technological approaches, offers block-specific insights, and discusses their clinical practice indications, such as postoperative pain management, as well as emergency physicians’ use of these techniques to treat severe conditions like fractures, trauma, and muscular injuries, or even as an adequate substitute for procedural sedation. UGRA is also being used more frequently in obstetrics to relieve pain during labor and delivery because of its broad safety and effectiveness. Although UGRA increases block accuracy and safety, there are risks such as hematoma, nerve damage, and local anesthetic systemic toxicity (LAST). Careful dosing, monitoring, sterile technique, and preparedness to handle emergencies – including lipid rescue therapy for LAST – are all necessary components of safe practice.

On the other hand, artificial intelligence (AI), simulation, portable ultrasonography, augmented reality, and robotics are improving UGRA by enhancing accuracy, safety, training, and access, and making operations more effective and accessible. The implementation of UGRA is constrained by institutional hurdles, costs, and skill requirements; consistent training and credentialing are essential to increase proficiency and safety. UGRA promotes healing, reduces opioid use, and improves pain management. Although there are risks, safety and accessibility are enhanced by careful technique, monitoring, and advances such as portable ultrasonography and AI. Standardized training is necessary for optimal use.

## Introduction

### History of regional anaesthesia in clinical practice

Before the introduction of anesthesia, operations were performed on conscious patients who would undergo severe pain, forms of physical restraint, and rapid operative techniques to substitute for the lack of analgesia. This led to high rates of mortality due to bodily reactions (such as increased vagal reflexes and sympathetic surges) toward the severe levels of pain. The use of anesthesia in medicine can be traced back to its first proper use in 1846, when Dr. William Morton used ether and nitrous oxide at Massachusetts General Hospital for dental purposes^[^1^]^. This revolutionary breakthrough in painless surgery has forever shaped the future of medicine, providing patients with a pleasant, seamless experience during invasive procedures. This has not only increased quality of life but also broken down the stigma and fears around pain during such procedures, which has increased patient engagement.

HIGHLIGHTS

- Ultrasound-guided regional anesthesia (UGRA) provides real-time needle guidance, improving accuracy and safety.
- Block techniques span fascial, peripheral nerve, and neuraxial applications.
- UGRA enhances postoperative analgesia and reduces opioid requirements.
- Risks include nerve injury, hematoma, and local anesthetic systemic toxicity, and these require strict safety steps.
- AI, AR, simulation, and portable ultrasound are advancing UGRA training and use.

There are two forms of anesthesia commonly used in practice: general anesthesia (GA) and local anesthesia (LA). GA is defined by the American Society of Anesthesiologists (ASA) as a drug-induced loss of consciousness during which patients are not arousable, not even by painful stimulation^[^2^]^. This differs from LA, which is more localized – it can be further extended to regional anesthesia (RA), which produces larger areas of anesthesia. Harvey Cushing coined this term in 1902; the field has since developed various techniques, such as neuraxial anesthesia (spinal, epidural, and caudal), peripheral nerve blocks (PNBs), and plexus blocks. This study will specifically focus on techniques associated with RA.

### The paradigm shift introduced by ultrasound guidance

To ensure precision, regional anaesthesia (RA) is used alongside imaging techniques such as ultrasound (US) guidance. US is a non-ionizing imaging technique that uses sound energy in the spectrum, transmitted through a medium as a mechanical wave^[^3^]^. This is a diagnostic test used to confirm pregnancy. The adoption of US in regional anesthesia (USGRA) has marked a true paradigm shift, with direct visualization of nerves and landmarks improving the efficacy and safety of anesthetic practice. Before the use of US, clinicians would use blind techniques, which were often landmark-based and included paresthesia techniques; this posed safety risks, such as increased risks of nerve injury and vascular puncture. This also required higher volumes of anesthesia due to low placement precision, which substantially increased the risk of local anesthetic systemic toxicity (LAST). After its first use in 1996, it was observed that USGRA was associated with better onset times for anesthesia, longer block durations, and higher precision in identifying nerves – specifically the femoral, sciatic, and forearm nerves – which are major nerves commonly targeted. In addition to increased precision in nerve identification, it also allowed the observation of adjacent anatomical structures, which helped prevent damage to nearby structures and minimized the overall risk of complications^[^4^]^.

### Current relevance in perioperative and emergency care

There is a call to increase training in the implementation of USGRA among physicians in emergency care. This is due to the significant role that USGRA plays in emergency care. It is commonly used to treat trauma-induced long bone fractures via a PNB; this also decreases the utilization of opioid medications, which carry associated risks of complications such as respiratory depression and disturbances of consciousness. US guidance has enhanced the safety and reliability of such procedures^[^5^]^. It is also used in the management of pain from hip fractures and clavicle fractures, as well as in procedures such as reduction of shoulder dislocations, abscess drainage, and tube thoracostomy, with a low risk of neurapraxia or LAST compared to standard practices^[^6^]^.

In addition to its use in emergency care, it is used for peri-operative purposes. It is famously used in cardiac, thoracic, and breast surgeries, such as mastectomy, via the erector spinae plane block (ESPB) to reduce opioid consumption after surgery and intra-operatively. This procedure consists of a US-guided injection of local anesthetics into the region between the transverse processes of the vertebrae (between T2 and L2) and the erector spinae muscles. ESPB is crucial for pain management, particularly for neuropathic pain^[^7^]^.

### Objectives and scope of the review

This review will explore the foundations of USGRA and the classification of regional anesthetic blocks. It will further explore the technical approaches – for example, a block-by-block overview – and the indications for use in clinical practice. Toward the end, we will evaluate the clinical outcomes of USGRA through analyses of key studies and meta-analyses. As with every fast-emerging topic, there are common barriers to implementation and challenges it may bring – for example, ethical and financial considerations – which we will outline and for which we will propose possible measures.

## Foundations of ultrasound-guided regional anesthesia

### Core US principles for nerve visualizations

US works on the principles of sound transmission. Sound can travel through different materials by interacting with particles within a vibrating medium; its frequency is measured in hertz, a key parameter employed in ultrasonography. It is a form of non-ionizing radiation that corresponds to the acoustic portion of the spectrum and is transmitted through a medium as a mechanical wave. The higher the frequency, the better the image resolution. The image is generated by the exchange of electrical and acoustic energy within the piezoelectric crystals in the US probe. These crystals convert electrical energy into sound waves, which are reflected back to the probe. The probe captures these reflected waves.

### Equipment essentials: transducers, probes, and needles

To perform a USGRA, it is essential to use appropriate equipment depending on the circumstances. A transducer plays a key role in US scanning; it generates and receives the energy used to produce the image. There are two main types of transducers: low-frequency and high-frequency. Low-frequency transducers are used to increase US penetration (depth), which is useful for imaging deeper organs, such as the kidneys. High-frequency transducers focus on improving definition, which helps overall image clarity, and are often used to scan superficial structures (e.g., the thyroid, muscles, and vessels). As the frequency increases, the wavelength decreases, which is advantageous because it provides better distinction between a target and other objects^[^8^]^. A US transducer should be chosen depending on the depth of the targeted nerve or fascial plane^[^9^]^.

An adequate selection of probes is essential as well. There are three types of probes: linear, curvilinear, and phased array. Each probe has considerable advantages and is tailored to the situation. The linear array has a rectangular scanning area; the curvilinear array uses a curve to generate non-parallel waves and a cone-shaped scanning area; and the phased array has a small footprint with a large base to optimize the field of view. Specifically, echogenic short-beveled needles are recommended if available; otherwise, other short-beveled needles, blunt-tip spinal needles, or Tuohy-tip needles are also acceptable. The needle length should be directly related to the depth of the structures to be anesthetized. Alterations can be made to the needle tip to enhance sonographic recognition, for example, by adding a piezoelectric crystal to create vibration at the tip.

Needle guides are also attached to the US probe to maintain needle stability within the plane. The use of guidance reduces the time required to perform supraclavicular nerve blocks, femoral nerve block (FNB), or catheter placement, and spinal punctures. Lasers are commonly used to aid needle alignment by projecting a laser beam onto the corresponding US plane on the skin surface; however, this can leave the plane after the needle has penetrated the skin due to the beam’s superficial projection^[^10^]^.

### Understanding sonoanatomy: nerves, muscles, and vascular landmarks

Understanding sonography is essential in USGRA, as it helps the transition from inference to targeted visual practice, thereby improving overall success rates and reducing complications. Recognition of nerve appearance and fascial planes improves block success and lowers the number of needle passes^[^11^]^.

Peripheral nerve sonographic appearance in the cross-sectional axial view is often described as a honeycomb pattern due to the bright epineurium and perineurium surrounding fascicles, a brightness caused by densely packed connective tissue. In the long-axis view, the nerve appears as an elongated structure with alternating hypo- and hyperechoic bands^[^12^]^. The nerves are distinguished by their low anisotropy, meaning there is minimal alteration in echogenicity with changes in transducer angle. This is helpful when comparing nerves with tendons and muscles^[^13^]^. Vessels can be differentiated from nerves by the presence of pulsatile movement or collapsibility under pressure (vessels collapse, whereas nerves do not). Collapsibility is assessed with gentle probe compression^[^14^]^. Muscles appear striated and moderately hyperechoic, with hyperechoic perimysial septa in the long-axis and punctate echogenic dots in the short-axis.

### Comparative advantages over traditional approaches

Using USGRA has significant advantages over traditional techniques, particularly regarding patient safety and the reduction of the risk of complications. This technique uniquely provides live, real-time needle trajectory tracking, which reduces the risk of trauma to adjacent structures, including nerves, vessels, and the pleura^[^15^]^. Compared to blind techniques, US guidance allows physicians to inject regional anesthetic at precise points along the course of a nerve to achieve a specific endpoint; for example, a US-guided adductor canal/femoral triangle block reduces the incidence of quadriceps weakness following total knee arthroplasty (TKA)^[^16^]^.

US guidance also helps identify fascial planes to aid local spread into the correct plane. It is associated with a reduced incidence of vascular injury, dose-related side effects, pneumothorax, and phrenic nerve block, which can cause respiratory complications such as diaphragmatic paralysis.

## Classification of regional anesthetic blocks

RA is classified as upper limb block (ULB; blocks for the arm and hand), lower limb block (LLB; blocks for the leg and foot), neuraxial block (injections around the spinal cord for broad lower body anesthesia)^[^17^]^, and fascial truncal block (injections targeting specific tissue planes in the trunk and face)^[^18^]^ (Fig. 1).

**Figure 1.**
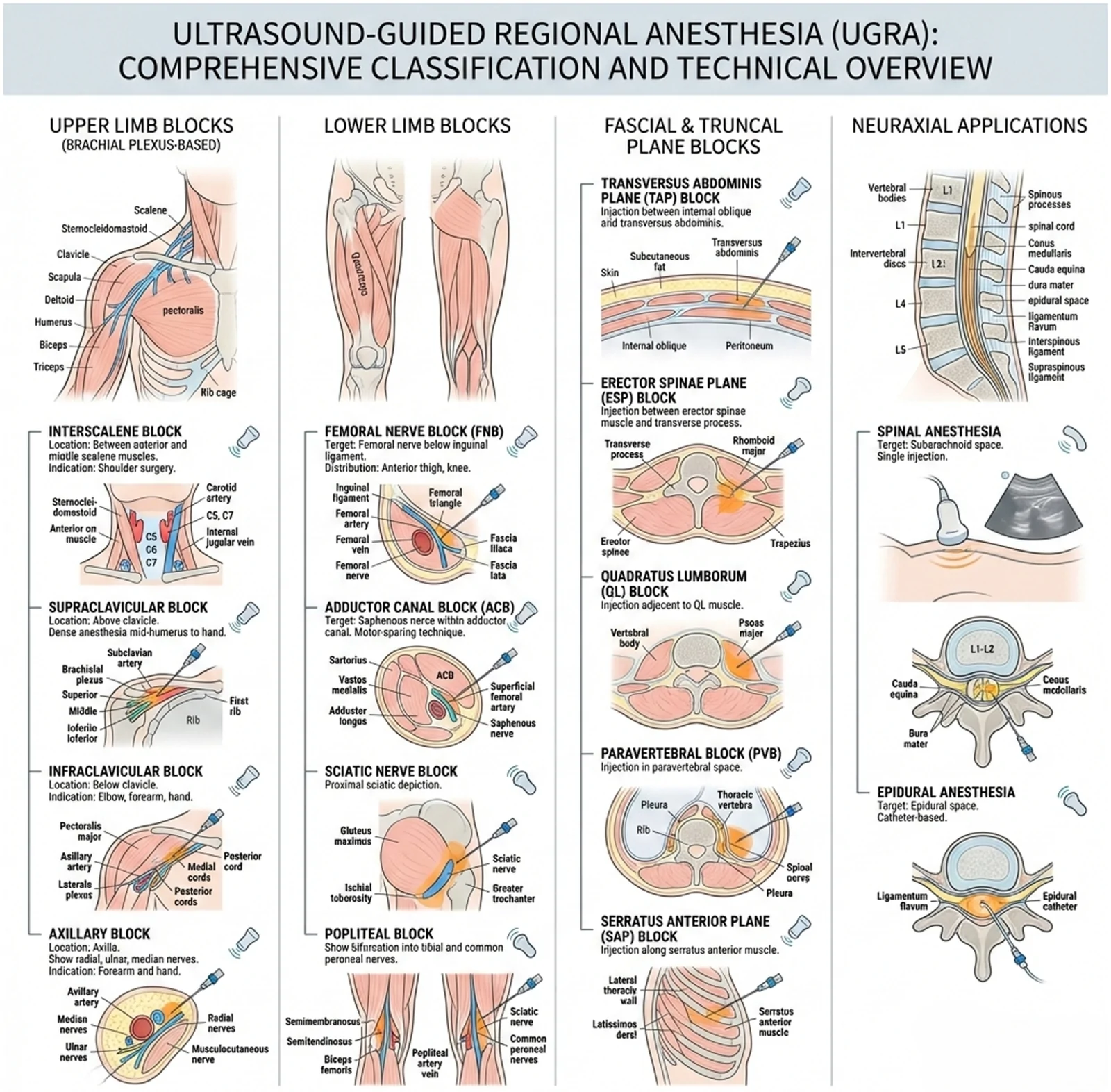
Comprehensive classification of ultrasound-guided regional anesthesia (UGRA). This schematic illustration summarizes the contemporary classification and anatomical distribution of UGRA, organized into four principal categories: upper limb blocks, lower limb blocks, fascial and truncal plane blocks, and neuraxial applications.

### Upper limb blocks

When performing upper-limb procedures, a common regional anesthetic approach is a brachial plexus block. A brachial plexus block can be performed using four basic approaches: axillary, supraclavicular, infraclavicular, and interscalene^[^19^]^.

#### Interscalene block

It is the most widely used postoperative analgesic technique for shoulder surgeries^[^20^]^. (analgesic means pain-relieving). It should be differentiated from anterior scalene muscle block (ASMB), a nerve block used for diagnosis and therapy in nerve-related conditions. Braun *et al* (2015) demonstrated that ASMB temporarily relieved neurovascular compression (pressure on blood vessels and nerves) through targeted muscle relaxation and may assist in the diagnostic evaluation of neurogenic thoracic outlet syndrome (a condition causing nerve symptoms in the arm), potentially supporting the decision to proceed with surgical intervention^[^21^]^.

#### Supraclavicular block

First described by Kulenkampff in 1911, this technique provides dense anesthesia for surgeries from the mid-humerus to the hand. However, the risk of pneumothorax led to its decline in use. As D’Souza and Johnson (2023) noted, the advent of US has significantly improved safety and accuracy, leading to renewed clinical interest in this technique^[^22^]^.

#### Infraclavicular block

Although it is not a reliable method for shoulder coverage, the infraclavicular block is frequently used for elbow, forearm, and hand surgeries because it anesthetizes the brachial plexus. Williams *et al* (2023) emphasized that this approach was developed to overcome the limitations of the supraclavicular block, particularly the risk of pneumothorax, and is widely recognized as a safe and effective alternative^[^23^]^.

#### Axillary block

It targets the neurovascular bundle’s median, ulnar, and radial nerves, which are the brachial plexus’s terminal branches. The method offers dependable anesthesia for the hand, forearm, and elbow. In addition, it provides cutaneous anesthesia of the inner upper arm, which is suitable for procedures requiring a tourniquet. Overall, this approach is considered the safest because of its lowest risk of serious complications^[^24^]^.

### Lower-limb blocks

For targeted anesthesia during hip, knee, ankle, or foot surgery, lower-extremity nerve blocks are helpful. The popliteal block, sciatic block, adductor canal block (ACB), and FNB are common techniques^[^25^]^.

#### Femoral nerve block

A local anesthetic is administered around the femoral nerve during a FNB, which reliably relieves pain in the anterior thigh, knee, and medial aspect of the calf, ankle, and foot, as highlighted by Liu *et al* (2022) and Murray *et al* (2010)^[^26,27^]^. A study found that FNB is widely used in patients with hip fractures because of its well-documented opioid-sparing effects and consequent reduction in opioid-related adverse effects such as nausea, vomiting, and delirium^[^28^]^. Despite these benefits, continuous FNB is associated with certain challenges, including catheter dislodgement, catheter-related infections, and quadriceps muscle weakness, which may impair postoperative mobilization and rehabilitation^[^27^]^.

#### Adductor canal block

ACB has gained prominence in TKA as a motor-sparing alternative to the FNB. It is reported that ACB provides pain relief equivalent to FNB while minimizing quadriceps weakness. Expanding on this, Agarwal *et al* (2025) conducted a scoping review and highlighted ACB’s consistent opioid-sparing effect, along with its positive impact on functional recovery and early mobilization^[^29,30^]^. Despite these advantages, when delivered via a continuous catheter, ACB is associated with substantially higher costs, requires greater technical proficiency, and is vulnerable to catheter dislodgement or migration – factors that can compromise the consistency of analgesia^[^13,29^]^. Although infection may not increase proportionally, catheter-based delivery introduces risks of bacterial colonization and persistent neuropathic symptoms – including long-term saphenous nerve neuropathy in a subset of patients – that warrant careful consideration, as noted by Ho *et al* (2025) and Agarwal *et al* (2025)^[^30,31^]^.

#### Sciatic and popliteal nerve block

Sciatic nerve block is used for orthopedic procedures involving the lower limb. According to Abd-Elfattah *et al* (2023), there are two approaches: the parasacral approach for sciatic nerve block may be associated with complications, while the popliteal approach offers safer and more effective analgesia^[^32^]^. Anatomical studies have confirmed variability in the sciatic nerve branching into the tibial and common peroneal nerves within the popliteal fossa. For diabetic patients in particular, the popliteal block, which targets the sciatic nerve in the popliteal fossa, is an excellent anesthetic option for foot and ankle procedures^[^33^]^.

### Fascial and truncal plane blocks

Fascial plane blocks (FPBs) provide pain relief for surgeries of the trunk, including thoracic, breast, and abdominal procedures. Truncal regional techniques are considered the gold standard for managing acute postoperative pain due to their superior analgesic effects. Research has demonstrated that, among the various FPB methods, truncal FPBs – such as the serratus anterior plane block, transversus abdominis plane block, quadratus lumborum block (QLB), ESPB, and paravertebral block (PVB) – are effective techniques^[^18,34^]^.

#### Transversus abdominal plane

Extensively described by Tsai *et al* (2017) and later reviewed by Tran *et al* (2019), this technique involves the deposition of a local anesthetic between the internal oblique muscle and transversus abdominis muscle^[^35^]^, where thoracolumbar nerves (T6–L1) traverse, providing adequate analgesia for abdominal surgery^[^36^]^.

#### Erector spinae plane block

A meta-analysis by Viderman *et al* (2022) demonstrated that it is a technique performed by administering a local anesthetic into the plane located between the erector spinae muscle and the thoracic transverse processes. It blocks the transmission of nociceptive stimuli through the dorsal and ventral rami of the spinal nerve roots, prevents the transmission of afferent stimuli, and inhibits efferent activation of the sympathetic nervous system^[^37^]^.

#### Quadratus lumborum block

The QLB is a recently developed FPB in which anesthetic is injected near the QL muscle (a muscle of the lower back). After hip replacement, the QL block has been linked to notable reductions in postoperative nausea, vomiting, pain, and narcotic use^[^38^]^.

#### Paravertebral block

First described in 1905, the PVB was re-examined as a truncal regional anesthetic in 1979, following multiple investigations that described it as a catheter-based approach^[^18,39^]^. A randomized controlled trial by Moorthy *et al* (2021) demonstrates that it lowers postoperative pain and opioid requirements when compared to systemic analgesia. PVB has been used extensively for analgesia following thoracic surgery for more than 20 years. PVB is regarded as the “gold standard” of thoracic regional analgesic techniques, along with epidural analgesia^[^39^]^.

### Neuraxial applications: ultrasound-assisted spinal and epidural techniques

Neuraxial anesthesia – spinal and epidural – remains the cornerstone of modern practice, with several studies highlighting that spinal anesthesia is typically simpler and faster to initiate. In contrast, epidural anesthesia permits titration and prolonged analgesia but is technically more demanding^[^24,40^]^. Many studies report that integrating US into neuraxial practice reduces the rate of failed or traumatic punctures and decreases the number of needle passes. It improves overall success, particularly when used pre-procedurally to identify the interspace, midline, depth to the epidural or subarachnoid space, and an optimal needle trajectory^[^40,41^]^. Although US guidance was described decades ago, wider adoption followed improvements in imaging during the early 2000s^[^42^]^.

Consistent with these findings, several studies and expert sources suggest that real-time US guidance is feasible but not routinely used due to practical challenges with needle advancement during simultaneous scanning^[^41^]^. Many studies also emphasize the added value of pre-procedural scanning in difficult scenarios – such as obesity, pediatric patients, spinal deformity, or when precise puncture site selection is critical – where it can meaningfully enhance accuracy, efficiency, and patient outcomes^[^41,42^]^.

## Technical approaches: a block-by-block insight

### Upper limb block

ULBs are indicated for surgery of the upper limb in children, adolescents, and in trauma cases to reduce pain, provide postoperative analgesia, and prevent chronic pain syndrome. Before performing a ULB, clinicians should carefully assess the risks, benefits, and alternatives to ensure patient safety^[^43,44^]^. US techniques use a linear probe for optimal visualization, and catheter-based continuous infusions are used for brachial plexus blocks. For needle selection, Pester *et al* (2025) recommended a short-beveled needle^[^22,23,24,25^]^ with gauge and size depending on patient age^[^44^]^.

The sterile US probe is placed in the supraclavicular fossa in a transverse orientation, caudally directed toward the thoracic cavity to visualize the brachial plexus near the subclavian artery. Precise patient positioning is essential for ULB. The general approach is to place the patient supine, with the head and neck rotated slightly toward the contralateral side; variations may apply depending on the specific block being performed. For the interscalene approach, the ipsilateral arm should be adducted. In contrast, for infraclavicular and axillary approaches, the arm may be abducted to 90 degrees with the elbow flexed^[^44^]^. An extensive cohort study by Zadrazil *et al* (2020) suggests that no major complications occurred in children^[^43^]^. However, another study notes that complications may still occur, including neuropraxia, vascular injury leading to hematoma, LAST, pneumothorax, phrenic nerve palsy, and Horner’s syndrome^[^44^]^.

### Lower limb block

LLBs provide targeted anesthesia for hip, knee, ankle, or foot surgeries, as reported by Prasad *et al* (2024)^[^25^]^. Furthermore, they are frequently utilized postoperatively to improve pain control, lower the acute postoperative opioid burden, provide opioid-sparing effects, facilitate early mobilization, and enhance outpatient feasibility. Depending on the extent of anesthesia required for the procedure, the lower extremity can be anesthetized by blocking nerves individually or in combination. For needle selection, an 18–20 G needle is used for drawing up local anesthetics, an 18–20 G needle is used, and adjuvants such as dexamethasone or dexmedetomidine may be utilized. Precise needle placement under US guidance is essential for patient safety^[^45^]^.

According to Rodziewicz *et al* (2025), complications are few, and the procedure should be avoided in patients with hypercoagulable states, thrombocytopenia, or pre-existing skin infections. Local complications include hematoma, nerve injury, and local anesthetic systemic toxicity. In contrast, block-specific adverse effects include quadriceps weakness with the FNB and an increased risk of falls after the fascia iliaca block. Overall, LLBs provide adequate analgesia, facilitate early mobilization, reduce hospital stays, and improve patient satisfaction^[^45^]^.

### Fascial plane block

FPBs have shown promise in the management of chronic pain. In a recent study, Ní Eochagáin *et al* (2024) showed that the truncal fascial plane has shown promise in improving recovery while reducing reliance on systemic medications and opioids^[^18^]^. The fasciae are typically visualized as thin, hyperechoic lines encircling the muscles and nerves, with a tiny amount of hyperechoic adipose tissue between the layers of fascia. This interventional technique is typically performed under US guidance. Marrone *et al* (2024) highlight that FPBs can be performed using both in-plane and out-of-plane techniques; however, in-plane needling offers a distinct advantage by providing visibility of the needle from the tip along its full length^[^46^]^.

Despite these benefits, FPBs carry specific risks. As Werry, Uppal, *et al* (2024) noted, potential risks vary with the anatomical site of injection and may include hemorrhage, liver or intestinal damage, peritonitis, pneumothorax, and local anesthetic toxicity. Careful technique and thorough anatomical knowledge are therefore essential to minimize these hazards^[^47^]^.

### Neuraxial block

Neuraxial blocks are recommended for revascularisation surgeries. They are also recommended in difficult scenarios in which palpating landmarks may be challenging, such as in elderly or obese patients, or those with spinal deformities^[^41,42^]^. From a technical perspective, curved-array US probes with low frequency (e.g., 2–5 MHz) are typically employed. Adjusting the depth (often 7–10 cm), focus placement, and gain parameters on the US machine are essential for optimizing sonographic images. Yoo *et al* (2020) emphasized the importance of preparation, particularly the removal of gel or chlorhexidine before needle insertion, to prevent complications such as arachnoiditis. According to the study, US guidance improves success rates and reduces complications. Possible side effects of a lumbar neuraxial block include painful needle placement, repeated punctures, and accidental dural puncture^[^48^]^.

Recently, advances in technology have enabled real-time US-guided spinal anesthesia to be performed using an electromagnetic needle-tracking system. These tracking systems help prevent damage to the conus medullaris, which can result from an accidental dural puncture at the level above the L1–L2 interspace^[^48^]^.

## Clinical indications and expanding applications

### Postoperative pain management

Since rapid recovery with improved physiological function after surgery is essential for good quality of life, it mandates post-operative care plans including physiotherapy, exercises, and multimodal analgesia, where UGRA has become a pivotal approach in various surgical disciplines. Combining UGRA with other pain-killers like non-steroidal anti-inflammatory drugs (NSAIDs) can create an impactful and comfortable pain-relief strategy for patients, thus facilitating early rehabilitation by enhancing pain control in ambulatory settings^[^49^]^. Compared with conventional landmark-based nerve stimulation (NS) techniques, UGRA has been shown to be superior in terms of efficacy, block accuracy, and rapid onset. In an randomized controlled trial (RCT) of 188 patients undergoing axillary brachial plexus surgery, comparing UGRA with NS alone, the block success rate was higher with UGRA (80.7%) than with NS alone (62.9%). Moreover, fewer adverse effects, including systemic toxicity, pain, and bruising, were reported with UGRA, most likely due to the lower local anesthetic volume used and accurate needle placement under US guidance^[^50^]^.

UGRA is a novel approach to address post-operative pain in different surgical settings. It can effectively reduce postoperative pain scores and opioid requirements by providing targeted analgesia. In orthopedic surgeries, UGRA is crucial to control post-operative pain, as it involves complex, often extensive and invasive procedures that require dissection and fixation of complex fractures and joint replacements^[^51^]^. A recent retrospective study on 533 patients reported that patients who received PNBs had significantly lower post-operative opioid use than those who did not (32.8% vs 61.9%)^[^52^]^. The use of PNBs in orthopedic surgery has increased substantially due to their favorable safety profile and adequate post-operative analgesia when combined with UGRA.

Additionally, they can be utilized for nerve blocks in procedures such as knee or hip replacements^[^53^]^. Moreover, a systematic review and meta-analysis comprising 849 cardiac surgery patients evaluated the effects of UGRA compared with placebo on post-operative opioid intake during the first 24 hours after cardiac surgery. Interestingly, the results favored UGRA, as all types of UGRA, including fascial block, erector spinae block (ESB), and pectoralis nerve block I, demonstrated a significant reduction in post-operative opioid requirements, thereby shortening the time to extubation and the duration of hospital stay^[^54,55^]^.

### Emergency medicine and trauma settings

Moderate to severe pain is one of the main reasons patients present to emergency departments (EDs). However, its effective and safe management remains a challenge. UGRA provides rapid pain relief via direct nerve blocks and reduces opioid use and its side effects^[^56^]^. UGRA has broadened horizons in emergency medicine (EM) and trauma management protocols as well. Emergency physicians (EPs) integrate it into their practice for addressing harrowing conditions such as muscle injuries, trauma, and fractures, and it can even serve as an alternative to procedural sedation^[^57^]^. It is effective in managing musculoskeletal pain because of accurate local anesthetic injection guided by US imaging and visualization of the nerves^[^58^]^. Beyond this, UGRA also has a role in treating visceral pain and managing ventricular arrhythmia, a cardiac emergency^[^59^]^. The American College of Emergency Physicians emphasized the role of UGRA in EM and trauma settings as an integral part of patient management in the ED, in accordance with ATLS protocols^[^60^]^. However, despite recognition of the benefits of UGRA in the ED, its use varies among clinicians. There is support for multidisciplinary integration, including anesthesiology and EM, to develop guidelines for its applications and to increase access while maintaining quality and standards. However, this approach must be implemented quickly, as delays in planning may limit patient access to UGRA innovations and benefits^[^61^]^. UGRA has become easier and more feasible to apply due to the advent of point-of-care US^[^56^]^.

### Obstetric and pediatric analgesia

Due to its proven safety and efficacy, UGRA is increasingly used in obstetrics for pain relief during labor and delivery. This approach has a high success rate due to direct US-guided visualization of anatomic structures and precise needle placement for RA^[^62^]^. In obstetrics, epidural and spinal analgesia are typically preferred for pain relief during labor and delivery. Nevertheless, UGRA is an innovation that has sparked questions about potential side effects during labor, necessitating further studies for thorough evaluation^[^63^]^. Systemic analgesia is an alternative to neuraxial techniques but is avoided due to substantial side effects; however, it is recommended that the process be completely monitored if systemic opioid analgesia is chosen. Lumbar peridural anesthesia and combined spinal-epidural analgesia are the usual safe techniques for labor pain relief if there are no contraindications to them. In contrast, there is an increased risk of instrumental delivery with neuraxial analgesia; therefore, it should be initiated as early as possible, without requiring any particular cervical dilation^[^64^]^. In children, GA is usually used, but there is no option except RA in painful and invasive procedures when GA is not available. These cases may include children with chronic or emergency conditions who are at risk of aspiration^[^65^]^. The application of post-operative analgesia via epidural catheterization decreases the requirement for NSAIDs or opioids, thereby putting parents and staff at ease. With recent advances and innovations such as UGRA, RA is now considered much safer for children in emergency scenarios^[^66^]^. A recent systematic review and meta-analysis found that UGRA provided greater analgesic efficacy with fewer anesthesia-related adverse effects and reduced postoperative opioid use^[^67^]^.

### Day-care surgeries and enhanced recovery protocols

Furthermore, UGRA also contributes to better post-operative care through early mobilization, particularly in day-care settings and within the framework of Enhanced Recovery After Surgery (ERAS) protocols, including total hip arthroplasty and TKA. UGRA does so by providing early, targeted, and effective pain management and by decreasing anesthesia-associated side effects, such as postoperative nausea and vomiting (PONV)^[^68^]^. Through UGRA-mediated pain relief, patients can be discharged early, enabling them to ambulate and participate in physical therapy. Additionally, reducing postoperative opioid use, with the consequent decline in PONV, is a crucial component of ERAS protocols, as PONV and drowsiness can even delay discharge. Hence, UGRA is an integral component of fulfilling the aims and scope of ERAS protocols and day-care surgery. This strategy is quite helpful in facilitating faster recovery after ambulatory surgeries such as arthroscopy, hernia repair, and reconstructive plastic surgery procedures, which mandate early mobilization, minimal opioid use, and quick discharge^[^68,69^]^.

### Oncology and chronic pain interventions

Since UGRA provides US-guided LA through precise needle placement, US-guided radiofrequency can also be used in ablation procedures. This principle can be effectively applied in oncology and chronic pain management. Furthermore, UGRA provides rapid and effective pain relief after tumor resection procedures and helps spare opioid-related side effects by reducing opioid use in chronic pain management^[^53^]^.

## Evaluating clinical outcomes: what does the evidence say?

### Pain score reductions and opioid-sparing effects

The decline in pain scores and satisfactory opioid-sparing effects achieved with UGRA via different kinds of nerve blocks have been validated by various studies. Recent reviews and clinical studies support the finding of lower pain scores, even at other postoperative times, with UGRA compared to placebo. A systematic review and meta-analysis comprising 5614 patients evaluated postoperative outcomes with UGRA and showed reduced postoperative opioid requirements^[^70,71^]^. Another systematic review and meta-analysis assessing patients undergoing median sternotomy advocated for the use of UGRA in cardiac surgery patients perioperatively and demonstrated opioid-sparing effects as well as practical and rapid pain reduction. Among the different types of nerve blocks performed via UGRA, the ESB has been recognized as the most efficient^[^54,72^]^. There are numerous studies demonstrating the effectiveness of LA application via UGRA techniques, and the outcomes undoubtedly represent a turning point in pain management. However, more RCTs are required to properly define the volume, concentration, and application of LA^[^72^]^. Similar results have been shown in abdominal, truncal, and orthopedic surgery studies regarding post-operative pain score reduction^[^51^]^. Among fascial blocks, pecto-intercostal fascial blocks have been regarded as among the most effective for early extubation^[^73^]^.

### Impact on hospital stay and functional recovery

The current data support the use of interventions to address medical and surgical issues, as they are strongly associated with improved patient-reported outcomes and reduced hospital stays. These interventions effectively improve patients’ post-operative quality of life and enable early ambulation and timely rehabilitation. Enhanced recovery pathway (ERP) interventions in lower limb arthroplasty and colorectal surgeries have potentially reduced patients’ hospital length of stay (LOS); UGRA for post-operative pain management and emergency cases has also significantly reduced LOS^[^74^]^. Besides these benefits, this approach is even mandated in day-care surgeries, and UGRA is a practical addition to such procedures as hernia repair^[^69^]^. Of the 25,984 studies retrieved, 26 met the inclusion criteria. Recent developments in anesthetic drugs have improved safety and efficacy, reducing complications. Moreover, advanced monitoring devices, such as multiparameter and brain function monitors, have enhanced patient safety through real-time assessments. Innovations in RA and US-guided nerve blocks have opened new clinical avenues for improved pain management, reduced recovery time, minimized morbidity, and better patient-reported outcomes^[^75^]^. According to a narrative review, there are more favorable results with continuous PNBs in terms of minimized opioid consumption, higher patient satisfaction, and better analgesia, thus leading to decreased in-hospital LOS ^[^76^]^. According to a network meta-analysis, UGRA techniques reduce ICU stay and enable earlier extubation, thereby delivering desired perioperative results^[^54,55^]^.

### Patient satisfaction and improved mobility

Since ERPs are widely used to standardize perioperative care and outcomes, patient satisfaction with these ERPs, ERAS protocols, and UGRA-like innovations has significantly increased^[^77^]^. UGRA has also shown promising results in terms of patient satisfaction. In a cross-sectional study in Tanzania, 170 patients undergoing limb surgery with UGRA blocks reported satisfaction with this approach (95%). Hence, UGRA has high credibility in outsourced healthcare systems in low-income countries (Table 1)^[^78^]^.

**Table 1:** Summary of key studies and meta-analyses supporting UGRA.

	Scope	Evidence studies for UGRA
1.	Pain and opioid use	Reduction in post-operative pain Minimizes the perioperative opioid requirement Better perioperative management in both cardiac and non-cardiac surgeries^[^31,32^]^.
2.	Hospital stay and recovery	Reduced LOS Fulfilling the ERAS protocols for daycare surgeries^[^26,28,32^]^
3.	Patient satisfaction	High satisfaction Minimal or no adverse effects^[^29^]^
4.	Safety and chronic pain	Reduced post-surgical pain and prolonged opioid use at 3-6 months Management of chronic pain^[^33^]^.

**Table 2:** Summary of technique, needle use, US guidance, complications, and advantages of major blocks (upper limb blocks, lower limb blocks, fascial truncal block, and neuraxial blocks).

Block	Indications	Technique and needle	Ultrasound use	Complications	Advantages
Upper limb block	Upper limb surgery, trauma, pediatric cases, postoperative, and chronic pain^[^27,28^]^.	Linear probe; catheter-based technique; short beveled 22–25 G needle, usually depending on age^[^28^]^.	Probe in supraclavicular fossa best to visualize near subclavian artery^[^28^]^.	Neurapraxia, vascular injury, hematoma, LAST, pneumothorax, phrenic nerve palsy, and Horner syndrome^[^28^]^.	Good for intra- and postoperative analgesia, opioid sparing^[^27,28^]^.
Lower limb block	Hip, knee, ankle, foot surgeries; postoperative pain management^[^9,29^]^.	18–20 G needle used; block nerves^[^29^]^.	Essential for safety and accuracy^[^29^]^.	Hematoma, LAST, quadriceps weakness (femoral nerve block), increased risk of fall (fascia iliaca)^[^29^]^.	Effective analgesia and early mobilization, decreased hospital stay^[^29^]^.
Fascial plane block	Chronic pain, enhanced recovery, and opioid sparing^[^2,30^]^.	In-plane (preferred) or out-of-plane needle insertion^[^30^]^.	US visualizes fascia as hyperechoic lines^[^30^]^.	Hemorrhage, visceral injury, and peritonitis^[^31^]^.	Simpler, less invasive, and opioid sparing^[^2^]^.
Neuraxial block	Revascularization surgery; difficult anatomy (elderly, obese, and deformities)^[^25,26^]^.	Curved array probe (2–5 MHz); real-time tracking system; depth 7–10 cm^[^32^]^.	Essential for spinal landmarks and safety^[^25,26^]^.	Painful placement, repeated puncture, accidental dural puncture, and arachnoiditis^[^32^]^.	Increased success rate, decreased complications with US, useful in difficult cases^[^32^]^.

## Complications, pitfalls, and risk reduction strategies

### Common and rare adverse events (e.g., LAST, nerve injury, and hematoma)

UGRA is gaining popularity in EM, particularly in academic centers, as a strategy for effective analgesia. Although it was first described in the anesthesia literature, USGRA is now gaining traction in EDs. However, barriers such as limited procedural knowledge and safety concerns continue to hinder its broader adoption, particularly among EPs in both academic and community settings^[^56^]^.

Although RA under dynamic US guidance has grown in popularity across both anesthesiology and EM, safety concerns remain – particularly in ED settings, where EPs may have limited exposure to RA techniques. The use of large volumes of local anesthetics raises the risk of systemic complications such as LAST, a concern amplified in non-operating-room environments where immediate anesthesiology backup may not be available. It is known that local anesthetics work by attaching to the alpha subunit of voltage-gated sodium channels, even though the exact mechanism of LAST is not fully understood. A conformational shift induced by this interaction prevents sodium influx, thereby suppressing neuronal transmission and depolarization. In addition to inhibiting nerve conduction, local anesthetics decrease ATP synthesis, disrupt mitochondrial function, and alter cell membrane signaling. These substances have systemic effects across multiple organ systems: they inhibit the conduction of action potentials in nerves, they cause vasodilation in vascular smooth muscle, they decrease excitability and prolong the refractory period in the heart, and they initially trigger excitability in the central nervous system (CNS) before causing widespread depression. The complicated and varied clinical presentation of LAST, which can include both neurological and cardiovascular (CV) manifestations, is explained by the involvement of multiple physiological systems^[^79,80^]^.

A wide range of CV and CNS symptoms, either alone or in combination, are indicative of LAST. CV abnormalities often precede neurologic symptoms, although a fifth of patients may present with CV abnormalities first. Later features may include widened QRS complexes, sympathetic activation (e.g., tachycardia, hypertension, and diaphoresis), reduced contractility, which may primarily manifest as hypotension, and any type of cardiac arrest (ventricular tachycardia, ventricular fibrillation, PEA, or asystole)^[^81^]^.

While LAST remains the most feared systemic complication, peripheral nerve injury and hematoma also warrant consideration, particularly in high-risk populations. Complicated and multifaceted mechanisms frequently cause peripheral nerve damage during RA. Damage to the perineurium is thought to be the most critical element leading to nerve injury associated with PNBs, even if the total risk of permanent or severe nerve injury is extremely low^[^82,83^]^.

Hematoma is a known side effect of RA, especially for those on anticoagulant medications. The type and location of the block, along with the patient’s coagulation status, all affect the risk. The literature provides inadequate data on the hematoma risk associated with PNBs, despite its clinical significance. In response, the Canadian Anesthesiologists’ Society assembled an expert panel to categorize the risk of bleeding associated with different regional anesthetic procedures^[^80^]^. Although hematoma risk was a major issue, there was little clinical evidence to support most guidelines; therefore, they were based on expert consensus. These recommendations, which emphasize the value of a customized risk assessment when considering RA in patients at risk of bleeding, are intended as clinical guidance rather than a set norm.

### The role of US in minimizing risks

Proponents of UGRA highlight its ability to deliver more reliable nerve blocks, with higher success rates and better patient outcomes. UGRA permits the administration of lower local anesthetic doses, shortens procedure time and block onset time, and often results in longer block duration and greater patient satisfaction. Importantly, US enables physicians to visualize surrounding tissues, blood vessels, and nerves in real time, helping them avoid vital structures such as the pleura, lungs, and blood vessels. Complications such as arterial puncture, pneumothorax, nerve damage, and systemic toxicity associated with local anesthetics are less likely with this improved imaging. UGRA improves block quality, safety, and overall clinical efficacy compared with conventional landmark or NS approaches^[^84,85^]^.

### Protocols for safe practice and early detection of complications

The safe implementation of UGRA requires adherence to protocols designed to prevent, identify, and treat potential problems. While US improves safety, risks such as LAST, nerve injury, and hematoma cannot be eliminated. Therefore, a combination of patient selection, operator availability, and institutional protocols is necessary.

#### Pre-procedural safety measures

Perioperative treatment in regional anesthesia necessitates careful patient selection and preparation, since many patients continue to anticipate GA and may be anxious or hesitant about undergoing surgery using a regional block alone. Providing comprehensive preoperative information, such as instructional videos, reduces anxiety and improves patient satisfaction. Medication safety can be enhanced by labeling syringes, separating perineural and intravenous medications, and using two-person checks. During surgery, patients should receive adequate monitoring and comfort measures, with discharge instructions emphasizing the importance of protecting the insensate limb and providing effective analgesics to ensure a comfortable recovery^[^85^]^. Prior to initiating a regional anesthetic block, it is essential to ensure patient safety by using appropriate US probes, adhering to strict sterile technique, and performing real-time imaging of relevant anatomical structures. Standard monitoring in accordance with the ASA guidelines – including electrocardiography, pulse oximetry, and non-invasive blood pressure measurement – should be applied to detect early physiological changes during the procedure. Equally important is meticulous drug preparation: the choice, concentration, and maximum safe dose of local anesthetics must be carefully verified. Incremental administration with frequent aspiration not only facilitates accurate deposition of the anesthetic around the target nerve but also minimizes the risk of inadvertent intravascular injection and LAST, thereby enhancing both procedural safety and patient outcomes^[^86,87,88^]^.

#### Intra-procedural protocols

Real-time ultrasound guidance enables continuous needle-tip tracking, significantly reducing the risk of inadvertent vascular puncture and intraneural injection. Local anesthetic should be administered slowly in small aliquots (3–5 mL) with intermittent aspiration to detect any intravascular placement early and to minimize LAST. Adjuvants may be used to enhance block efficacy, but only when supported by evidence to avoid compounding systemic toxicity^[^87,88^]^.

#### Early detection of complications

Vigilant monitoring is required during and after regional anesthesia injection to detect early signs of complications. Clinicians should look for CNS signs of LAST, such as tinnitus, circumoral numbness, metallic taste, or agitation, as well as CV abnormalities, including tachycardia, hypertension, or arrhythmias. Regular post-block neurological tests should document motor and sensory function to detect any early nerve damage. To promptly diagnose compressive hematomas, patients on anticoagulation must be closely monitored for swelling, worsening discomfort, or new neurological impairments^[^89^]^.

#### Emergency management readiness

Ensuring preparedness for LAST is critical wherever UGRA is performed. Intravenous lipid emulsion should be readily available, and doctors must be trained in accordance with American Society of Regional Anesthesia (ASRA) standards for airway care, ACLS, and lipid rescue therapy. Immediate access to airway devices, defibrillators, and trained personnel is critical for successful resuscitation. Additionally, institutions should maintain complication registries to support reporting, auditing, and ongoing improvement in patient safety^[^90^]^.

## Innovations shaping the future of UGRA

### AI integration and automated nerve tracking

Artificial intelligence (AI) is revolutionizing UGRA by increasing anatomical target recognition, needle trajectory estimation, and tip localization, resulting in higher nerve block precision and safety. AI models, such as U-Net architectures and multimodal trackers, enable real-time recognition of nerves, fascial planes, and surrounding landmarks, minimizing reliance on operator experience while aiding non-expert users. Despite its potential, AI faces limitations such as anatomical heterogeneity, reliance on operators, and limited clinical acceptance, requiring careful interpretation by practitioners^[^91^]^.

UGRA demands optimal US image acquisition and interpretation to identify sonoanatomy, apply colour overlays, and guide needle placement and local anesthetic deposition, thereby reducing complications and increasing block success. AI can help with this process by emphasizing essential features such as blood vessels, enhancing safety, and serving as an educational tool for trainees to accelerate learning and build trust in sonoanatomy^[^92^]^.

By incorporating AI into clinical practice, UGRA can become safer, more efficient, and more accessible, allowing for personalized anesthesia approaches tailored to each patient’s anatomy while reducing problems. The technology assists both novice and experienced doctors by providing real-time advice and decision support, resulting in improved procedural accuracy, reduced risk, and better overall patient outcomes^[^93^]^.

### Simulation-based training and skills assessment

As UGRA is increasingly used in EM, it is critical to define the skills and abilities that EPs should acquire. Recent observations among Italian EM trainees indicated that UGRA education was rarely included in residency programs. To address this, a course was created that included basic knowledge from established UGRA courses, as well as hands-on training using *ex vivo* models such as turkey thighs, allowing students to practice needle-probe coordination and fascial-plane hydrodissection. Participants reported greater awareness of sonoanatomy and procedural confidence, but quick scanning sessions were insufficient to master anatomical variations^[^94^]^.

The implementation of structured training also requires addressing institutional and interdepartmental barriers, ensuring support from hospital leadership, and establishing defined pathways, such as hip fracture management, involving multidisciplinary teams. Despite limited evidence on the incremental effectiveness of simulation-based training, it remains useful for intentional practice, allowing learners to make and correct mistakes in a safe, controlled setting without jeopardizing patient safety^[^57^]^.

Modern training emphasizes gradual independence, integrating didactic teaching with observation and supervised engagement. Cognitive comprehension of the process, development of fundamental enabling abilities, and opportunity for practice across many platforms, such as virtual reality, bench models, cadavers, and animal models, are all important components of pre-patient training. By adopting structured, evidence-informed UGRA education, EPs can acquire competency, reduce procedural errors, and enhance patient safety while adapting to the growing complexity of procedural skills in contemporary clinical practice^[^95^]^.

### Portable US for low-resource settings

Portable point-of-care US (PPOCUS) devices have improved access to diagnostic imaging and UGRA in low-resource settings. These compact, cost-effective devices deliver high image quality while remaining inexpensive, sturdy, and easy to use, making them suitable for rural hospitals, emergencies, and remote clinics. Their portability eliminates the need for patient transfers, speeds up diagnosis, and enables faster interventions, which is especially significant in critical care and surgical situations.

The integration of AI with portable ultrasonography increases its utility by offering automated guidance, real-time image interpretation, and procedural support. AI algorithms can help recognize anatomical landmarks, suggest appropriate needle trajectories, and highlight essential structures such as nerves and blood vessels, thereby boosting procedural accuracy and safety, even when the operator has limited experience.

Furthermore, AI-enhanced PPOCUS facilitates telemedicine by enabling remote supervision, interpretation, and measurement of clinical data, allowing for specialist oversight in underdeveloped areas. Studies have shown that it may accurately diagnose conditions such as heart irregularities, pneumonia, and liver disease, as well as obstetric parameters, on a par with professional sonographers^[^96^]^.

Overall, portable US combined with AI technology bridges the gap in healthcare delivery in low-resource settings, enhancing the safety and effectiveness of UGRA and point-of-care diagnostics, and expanding access to essential care in areas with limited infrastructure^[^88,97^]^.

### Augmented reality and robotics in regional blocks

Robotics in anesthesia can be classified into pharmaceutical, mechanical, and cognitive systems. Pharmaceutical robots use feedback loops to optimize medication delivery; mechanical robots offer superior precision and dexterity; and cognitive robots provide decision support by suggesting or administering treatments. Robotic US-guided nerve blocks in RA have demonstrated high success rates, and robotic training methods shorten learning curves compared with traditional human-performed procedures. These technologies are crucial because they improve procedural accuracy, reduce complications, and help trainees learn complex techniques, ensuring patient safety^[^98^]^.

Advances in AI, augmented reality, and virtual reality enhance training and clinical performance. Systems such as Safer Injection automate aspiration and flow control, while motion analysis, eye tracking, and haptic feedback improve procedural skills and decision-making. AI-assisted networks assist in nerve recognition using US, and robotic systems may provide autopilot capabilities. Integrating robots and AI into augmented environments enables accessible, high-quality training and safer, more precise regional anesthetic procedures^[^99,100^]^.

### Summary of technique, needle use, US guidance, complications, and advantages of major blocks

## Barriers to implementation and clinical challenges

### Learning curve and operator dependency

One could consider UGRA a complex procedural endeavor that involves several areas, including ergonomics, professional qualities, hand-eye coordination, and theoretical understanding. Novices are taught the essential motor skill of real-time ultrasonography-guided needle placement into a target nerve. Some people find these tasks difficult, but others can readily visualize the three-dimensional mental image produced by moving the transducer in one hand while adjusting for the needle’s trajectory in the other. There is currently no known organized strategy for predicting a novice’s learning curve. The cognitive ability of humans to create, store, retrieve, manipulate, and comprehend visual information is known as visuospatial ability. Teachers and novices with poor visuospatial skills frequently feel anxious and frustrated. A novice with limited visuospatial ability has a longer, less steep learning curve than a novice with strong visuospatial ability. The worry and frustration that novices with inadequate visuospatial skills frequently feel can make it difficult for them to integrate RA into clinical practice^[^101^]^. Other learning approaches, such as practical clinical experience in RA, theoretical instruction on sonoanatomical information, and the management of procedural difficulties, cannot be entirely replaced by technology-enhanced training. Although each method may be more appropriate for specific aspects of RA performance, technology-enhanced learning holds great promise for improving RA training. Additionally, there is a need for technological development to enhance the availability, ease of use, accuracy, and functionality of devices^[^102^]^. The shortage of qualified anesthetists and physician anesthesiologists is a hurdle to the development of RA, particularly US-guided procedures. It is challenging to find and retain anesthesia practitioners because the field lacks sufficient support^[^103^]^.

### Equipment costs and institutional limitations

Expenditure on US technology and a shortage of qualified anesthesiologists have been identified as the main obstacles to the increased adoption of US treatments in low- and middle-income nations. In low- and middle-income nations, anesthesia capacity is further restricted by deficiencies in anesthesia infrastructure, supplies, and medications. The difficulties are significantly more noticeable in environments with limited resources^[^103^]^. Even where benefits are noted, institutional constraints dominate practice. In a survey of Southwestern Ontario anesthesiologists, the main barrier to the implementation of UGRA as routine practice was that 59% of survey participants reported restricted access to US machines, such as shared units across various operating rooms^[^104^]^. Other non-technical barriers included a lack of institutional support, for example, a lack of official policy or clinical leaders’ endorsement. To deliver comprehensive training in UGRA, a curriculum must clearly define the basics, select appropriate teaching methods, develop effective evaluation strategies, provide timely feedback, and maintain resident motivation and engagement. When training residents in invasive UGRA procedures, one must balance patient safety with many opportunities for hands-on experience. Simulation programs allow for safe and instance-based learning of anatomy and sonoanatomy, procedural skill set development, and decision-making outside of clinical scenarios. Furthermore, gamification (incorporating game-like elements such as leaderboards, challenges, and scores) can enhance safety, retention of skills, and interest among patients^[^105^]^.

### The need for standardized guidelines and credentialing

The ASRA and Pain Medicine recommend competency-based frameworks to ensure provider competence and to improve patient safety in UGRA practice. According to the evidence-based executive summary, UGRA education programs should provide a standardized curriculum that combines didactic instruction with practical training on ultrasonography equipment and supervised clinical practice. Second, they advocate official credentialing processes that entail assessing physicians and officially recognizing them as competent in UGRA before being permitted to perform the technique independently at their own institutions. These principles aim to improve overall block success rates, avoid technical issues, and reduce practice variance^[^106^]^

## Conclusion

Over the past 10 years, the use of US-guided PNBs has grown in popularity and has been documented for a variety of PNB types in both adults and children^[^107^]^. In this review, we explored UGRA stepwise according to the classification of different regional blocks. UGRA has become indispensable to contemporary pain management strategies. This technique facilitates early patient mobilization, curtails opioid consumption, and enhances postoperative recovery through targeted analgesic delivery, particularly in orthopedic, cardiac, and ambulatory surgical procedures; it delivers rapid, efficacious pain relief in emergency and trauma scenarios, thereby diminishing requirements for opioids and sedative agents; it elevates safety standards for obstetric and pediatric populations by minimizing pharmaceutical exposure while ensuring accurate needle positioning; it aligns with ERAS protocols; and it is gaining traction in oncology and chronic pain treatment owing to its precise neural targeting capabilities and reduced systemic adverse effects. Although UGRA improves safety and precision, there are potential complications associated with it, including hematoma, nerve injury, and LAST. By enabling precise, low-volume injections and real-time imaging of nerves and arteries, UGRA reduces these risks. Proper patient selection, sterile technique, incremental dosing with frequent aspiration, and ongoing monitoring are all essential components of safe practice. Staff training and standardized protocols also improve outcomes and reduce adverse events.

The precision, safety, and accessibility of UGRA are being enhanced by AI, simulation training, portable ultrasound, and robotics. These tools improve training outcomes, facilitate UGRA use in low-resource environments, and assist both experienced and inexperienced users. Clinical integration, operator dependence, and the lack of uniform training pathways continue to pose difficulties. Steep learning curves, expensive equipment, and a shortage of skilled personnel are obstacles to UGRA adoption. Training is influenced by institutional support and visuospatial abilities. Standardized protocols and certification form the foundation of safe, reliable practice. The future of UGRA ultimately rests on integrating innovation and education to increase accuracy, enhance patient outcomes, and broaden its application across a variety of clinical contexts.

TITAN Guidelines: This manuscript is in compliance with the TITAN Guidelines (2025), declaring no use of AI^[^108^]^.

## Data Availability

This narrative review is based on previously published literature. No new data were generated or analyzed in this study. Therefore, data sharing does not apply to this article.
